# Identification of Selected Physical and Mechanical Properties of Cement Composites Modified with Granite Powder Using Neural Networks

**DOI:** 10.3390/ma18163838

**Published:** 2025-08-15

**Authors:** Slawomir Czarnecki

**Affiliations:** Faculty of Civil Engineering, Wroclaw University of Science and Technology, Wybrzeze Wyspianskiego 27, 50-370 Wroclaw, Poland; slawomir.czarnecki@pwr.edu.pl

**Keywords:** cement composites, granite powder, neural networks, mechanical properties, machine learning

## Abstract

This study presents the development of a reliable predictive model for evaluating key physical and mechanical properties of cement-based composites modified with granite powder, a waste byproduct from granite rock cutting. The research addresses the need for more sustainable materials in the concrete industry by exploring the potential of granite powder as a supplementary cementitious material (SCM) to partially replace cement and reduce CO_2_ emissions. The experimental program included standardized testing of samples containing up to 30% granite powder, focusing on compressive strength at 7, 28, and 90 days, bonding strength at 28 days, and packing density of the fresh mixture. A multilayer perceptron (MLP) artificial neural network was employed to predict these properties using four input variables: granite powder content, cement content, sand content, and water content. The network architecture, consisting of two hidden layers with 10 and 15 neurons, respectively, was selected as the most suitable for this purpose. The model achieved high predictive performance, with coefficients of determination (R) exceeding 0.9 and mean absolute percentage errors (MAPE) below 6% for all output variables, demonstrating its robustness and accuracy. The findings confirm that granite powder not only contributes positively to concrete performance over time, but also supports environmental sustainability goals by reducing the carbon footprint associated with cement production. However, the model’s applicability is currently limited to mixtures using granite powder at up to 30% cement replacement. This research highlights the effectiveness of machine learning, specifically neural networks, for solving multi-output problems in concrete technology. The successful implementation of the MLP network in this context may encourage broader adoption of data-driven approaches in the design and optimization of sustainable cementitious composites.

## 1. Introduction

The production of cement, a crucial component in concrete, is a significant contributor to greenhouse gas emissions. The cement industry is responsible for approximately 7% of global CO_2_ emissions, primarily due to the calcination of limestone and the energy-intensive nature of cement manufacturing [1]. This environmental burden has prompted an urgent need for more sustainable practices within the construction sector. Consequently, there is growing interest in reducing the ecological impact of cement by incorporating alternative binders and replacing a portion of the cement used with supplementary cementitious materials (SCMs)—materials that can partially substitute Portland cement without compromising—and often improving—the performance of concrete [2].

SCMs are materials that, when used in conjunction with Portland cement, contribute to the properties of hardened concrete through hydraulic or pozzolanic activity. Common SCMs include the following: fly ash, a fine particulate residue produced during coal combustion in thermal power plants [3]; slag cement, derived from the rapid cooling of molten slag generated during iron production in blast furnaces [4]; and silica fume, a highly reactive ultrafine powder collected as a byproduct in the manufacturing of silicon metal or ferrosilicon alloys [5]. These materials are well established in concrete technology due to their beneficial effects on durability, strength development, and resistance to chemical attack, while simultaneously reducing the embodied carbon of cementitious systems [6,7].

In recent years, the scope of SCMs has expanded beyond conventional industrial by-products to include naturally occurring or waste-derived materials such as glass powders [8], quartzolite-rich materials [9], red mud [10], and granite powder [11], the latter having emerged as a promising candidate. Granite is a widely available igneous rock used predominantly in the construction and decorative stone industries [12]. When crushed and ground into a fine powder—often as a waste product from quarrying or stone-cutting operations—it can be repurposed in concrete applications, reducing landfill waste and promoting circular economy principles [13].

The potential of granite powder as an SCM lies in both its filler effect and latent pozzolanic activity, although the latter is generally weaker than that of more reactive materials such as silica fume [14,15,16]. Nonetheless, its physical characteristics (e.g., particle size and shape) can enhance particle packing in the cement matrix, improve workability, and potentially reduce porosity. Furthermore, the chemical composition of granite is a critical factor influencing its suitability as a cement substitute. Granite generally contains high proportions of silica (SiO_2_) along with alumina (Al_2_O_3_), alkalis, and other oxides, though these proportions vary significantly depending on geological origin [17].

For example, Lower Silesian granite, common in parts of Central Europe, is characterized by a relatively low quartz content, with silicon dioxide comprising just over 50% of its composition. In contrast, granite from Spain and China tends to have a higher SiO_2_ content, ranging from approximately 60% to 70% [18]. These differences not only affect the pozzolanic reactivity of the material, but also influence mechanical properties such as compressive strength, flexural strength, and shrinkage behavior of the final composite.

Beyond acting as a binder substitute, granite powder can also serve as a microfiller, refining the pore structure and reducing the water demand of cementitious mixtures. This dual functionality makes it an attractive option for enhancing both the mechanical performance and the sustainability profile of concrete.

Given that such changes in composition and microstructure are directly linked to the properties of the hardened material, there is a growing need to reliably model and predict performance. The introduction of new components such as SCMs, waste-derived powders, or nanomaterials introduces complex interactions at both micro and macro scales, which can significantly influence mechanical and physical properties, including strength development, shrinkage behavior, permeability, and durability. Accurate predictive tools are therefore essential to optimize mix designs and ensure structural reliability [19].

Unfortunately, existing standard methods and empirical equations—often based on conventional materials and outdated assumptions—are not always sufficiently accurate. In some cases, these models can deviate by up to 40% or more from experimental results [20]. This level of error can lead to unsafe designs or unnecessary over-dimensioning of structural elements, increasing material consumption and costs. The lack of flexibility and adaptability in traditional models limits their usefulness, especially for modern, complex concrete systems incorporating non-traditional materials.

In response to these challenges, recent research has increasingly turned to artificial intelligence (AI) and machine learning (ML) as powerful tools for performance prediction in cement and concrete technology [21]. Unlike traditional models, machine learning algorithms can identify hidden patterns in large datasets, capture nonlinear relationships between input variables, and generate accurate predictions without the need for explicit physical equations. These tools can adapt to new materials and unconventional compositions, making them particularly suitable for modeling the behavior of innovative cementitious composites.

Numerous studies have already demonstrated the successful application of ML algorithms in concrete technology. For instance, researchers have used machine learning to predict the compressive strength of concrete modified with nano-silica particles [22], the tensile strength of concrete incorporating metakaolin [23], the bond strength in reinforced concrete made with recycled aggregates [24], and the packing density of granular systems in composite mixtures [25]. The effectiveness of these models has been confirmed by high coefficients of determination (*R*^2^), low root mean square errors (*RMSE*), and other statistical indicators of model quality. These high accuracies illustrate the significant potential of machine learning to replace or complement traditional design approaches.

Despite these promising results, most existing applications of machine learning in this field focus on predicting a single property of concrete. While useful for academic demonstration or preliminary material screening, this approach is often insufficient for practical engineering purposes. When designing structural concrete elements, engineers must consider multiple properties to ensure safety, functionality, and economy. For example, density is essential for estimating the self-weight of structural elements, which affects load calculations and foundation design. Compressive strength defines the load-bearing capacity of concrete, while tensile strength is particularly critical in elements exposed to bending, cracking, and fatigue—such as slabs, pavements, and industrial floors.

Furthermore, the interdependence of concrete properties is often overlooked in single-output predictive models. This study addresses this gap by developing an integrated, multi-output modeling framework that simultaneously predicts compressive strength at 7, 28, and 90 days, bonding strength, and packing density. Such a comprehensive approach enables deeper insight into the synergistic behavior of cementitious systems, supporting more informed and optimized mix design decisions. Multi-objective modeling techniques—particularly those based on machine learning and hybrid AI approaches—are increasingly recognized as the next frontier in smart concrete technology. These tools provide the ability to evaluate complex trade-offs between key performance indicators such as strength, durability, cost, sustainability, and workability within a unified predictive environment.

## 2. Materials and Methods

**Figure 1:**
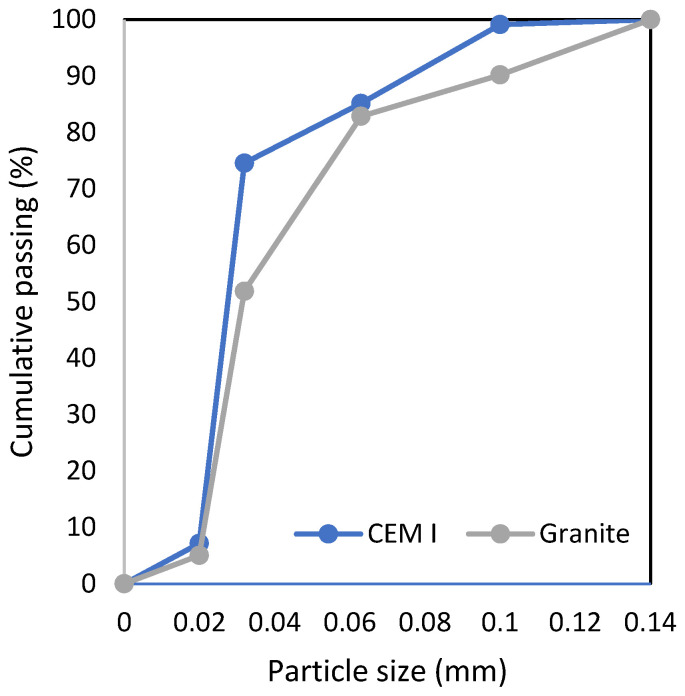
The sieve curve of cement and granite powder used in the study.

The samples used in this study were prepared and tested in strict accordance with applicable international standards and testing protocols, ensuring the reproducibility and reliability of the obtained results. All procedures related to mixing, casting, curing, and mechanical testing were conducted under controlled laboratory conditions. The cementitious composites designed for this research consisted of Portland cement CEM I 42.5 R, produced by Heidelberg Materials (Górażdże, Poland), in compliance with the EN 197-1 standard. As a partial replacement for Portland cement, granite powder was utilized. This powder was sourced as a by-product from the cutting and processing of granite rock in various small companies in Strzegom, Poland. The granite powder, obtained from waste generated during stone-cutting operations, was finely ground to achieve a particle size distribution comparable to that of cement, as illustrated in Figure 1. Its incorporation in the composite mixture not only promotes sustainable material utilization but also enhances packing density and improves the microstructural integrity of the final hardened product. The fine aggregate used in the composite mixtures was natural quartz sand, extracted from various deposits Kwarcmix (Tomaszów Mazowiecki, Poland). The sand was selected based on its high silica content, rounded grain shape, and low impurity level, which make it particularly suitable for high-performance cementitious composites. Prior to use, the sand was sieved and washed to ensure consistent particle size distribution and to remove dust and organic contaminants. The particle size distributions (also referred to as sieve curves) for both Portland cement and granite powder were determined using laser diffraction analysis and are presented in Figure 1; these are presented along with the chemical compositions of the materials in Table 1. The complete mix compositions for each variant tested in the study are summarized in Table 2. Each mixture was proportioned to investigate the influence of partial cement replacement with granite powder at varying substitution levels. The water-to-binder ratio (w/b), sand content, and curing conditions were kept constant across all samples to isolate the effect of binder composition. In total, four mix designs were prepared, allowing for a comprehensive analysis of the impact of granite powder on the fresh and hardened properties of the cementitious composites. Based on previous studies [26], the authors decided not to extend the substitution ratio beyond 30%, as higher replacement levels could negatively affect sustainability and economic performance indexes.

**Figure 2:**
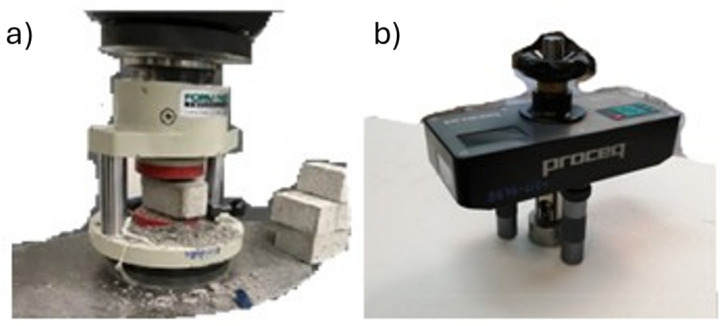
The view of the test stands for evaluating the (**a**) compression strength and (**b**) bond test.

Firstly, the physical properties of the fresh cement mixture—specifically its packing density—were evaluated before placing the material into the mold. Determining the weight of the material at this stage is crucial, as it is still in a fresh, non-load-bearing state. For each series, three measurements were conducted, resulting in a total of twelve tests. To comprehensively evaluate the mechanical performance of the cementitious composites developed in this study, a series of standardized laboratory tests were carried out on specimens prepared specifically for this purpose. All specimens were cast, cured, and tested in accordance with relevant international standards to ensure the reliability and repeatability of the results. The experimental program included the assessment of compressive strength and bonding strength (measured using the pull-off method), both of which are critical parameters for the structural application of cement-based materials. The compressive strength tests were conducted on prismatic samples with nominal dimensions of 40 mm × 40 mm × 160 mm. Specimens were demolded after 24 h and subsequently cured in water at a constant temperature of approximately 20 ± 2 °C. Prior to testing, the samples were weighed and measured to verify dimensional accuracy and to detect any irregularities that could affect the results. Testing was carried out after 7, 28, and 90 days of curing, with six samples evaluated for each time interval in each series. This allowed for the assessment of both early-stage strength development—which is important for formwork removal and construction scheduling—and long-term strength gain, which is essential for structural safety and service-life performance. In total, 72 samples were tested using a calibrated universal strength testing machine (shown in Figure 2) in compliance with EN 12390-3 [27]. In parallel with the compressive strength evaluation, the subsurface tensile strength of the composites was investigated using the pull-off adhesion test, a method commonly applied to assess the bonding and tensile properties of cementitious materials at or near the surface. For this purpose, cylindrical disc-shaped specimens with a diameter of 150 mm and suitable thickness were fabricated. This method was selected because it is particularly relevant for evaluating the mechanical properties of floors, where such composites might be used as an overlay. The pull-off tests were carried out in accordance with ASTM D4541-17 [28], using a portable pull-off strength tester equipped with a calibrated loading mechanism. The procedure involved bonding a metal dolly to the surface of the cured specimen with a high-strength adhesive, then applying a perpendicular tensile force until failure occurred. The maximum force required to detach the dolly was recorded and converted into tensile strength, expressed in megapascals (MPa). These tests were performed once, after 28 days of curing, with six samples evaluated per series, giving a total of twenty-four tested specimens.

**Figure 3:**
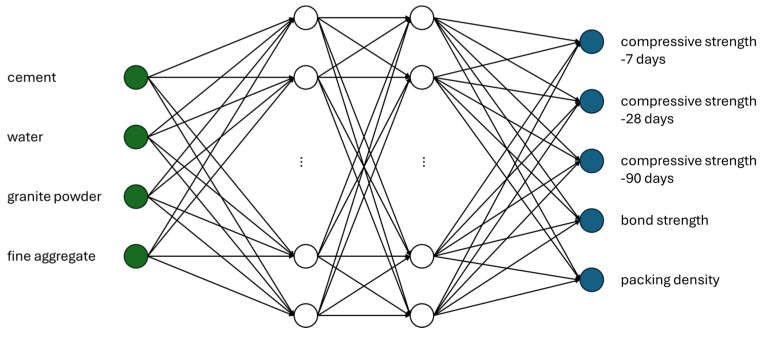
The scheme of neural network used in the study.

When predicting multiple dependent variables simultaneously, traditional statistical or regression-based models often fall short in terms of accuracy and flexibility. Among various machine learning algorithms, artificial neural networks (ANNs) have demonstrated exceptional performance, particularly in scenarios involving nonlinear relationships and interdependencies between outputs. These models can learn complex patterns within data, even in cases where traditional techniques struggle with overfitting or low generalizability. In this study, an ANN architecture was designed and implemented as the primary tool for modeling the behavior of the cementitious composites under investigation. The model was developed with four key input parameters, representing the most influential features derived from the material composition: cement content, granite powder content, water content, and fine aggregate content. The network architecture consisted of two hidden layers, enabling the model to capture the nonlinear interactions between input and output variables effectively. The output layer was configured to predict five distinct material properties simultaneously, including compressive strength, bonding strength, and packing density. A schematic representation of this multilayer, multi-output architecture is shown in Figure 3. To assess the robustness and reliability of the proposed network, additional models with a reduced number of output parameters were also developed and tested. These simplified models—predicting only one or two outputs—served as benchmarks for evaluating the trade-off between model complexity and predictive accuracy. This comparison provided insight into the network’s scalability and its ability to generalize to unseen data while maintaining computational efficiency. The performance of all models, both single- and multi-output, was evaluated using two widely accepted metrics: the coefficient of determination (*R*^2^) and the mean absolute percentage error (*MAPE*). *R*^2^ measures how well the predicted values correlate with the observed values, with 1.0 indicating perfect prediction. *MAPE* quantifies the average percentage deviation between predicted and actual values, providing a practical measure of prediction accuracy.

Both metrics were computed using the following mathematical formulas:

(1) $$R^{2}\;=\;\frac{\sum_{i\;=\;1}^{r}{\left(x_{i}-x_{mean}\right)}^{2}-\sum_{i\;=\;1}^{r}{\left(x_{i}-{\hat{x}}_{i}\right)}^{2}}{\sum_{i\;=\;1}^{r}{\left(x_{i}-x_{mean}\right)}^{2}},\;$$

(2) $$MAPE=\frac{1}{r}\sum_{i=1}^{r}\left|\frac{\left(x_{i}-{\hat{x}}_{i}\right)}{x_{i}}\right|*100,\;$$

where $x_{i}$ is the i-th actual value obtained in the experimental part, while ${\hat{x}}_{i}$ is the i-th value predicted by the network.

## 3. Results

**Figure 4:**
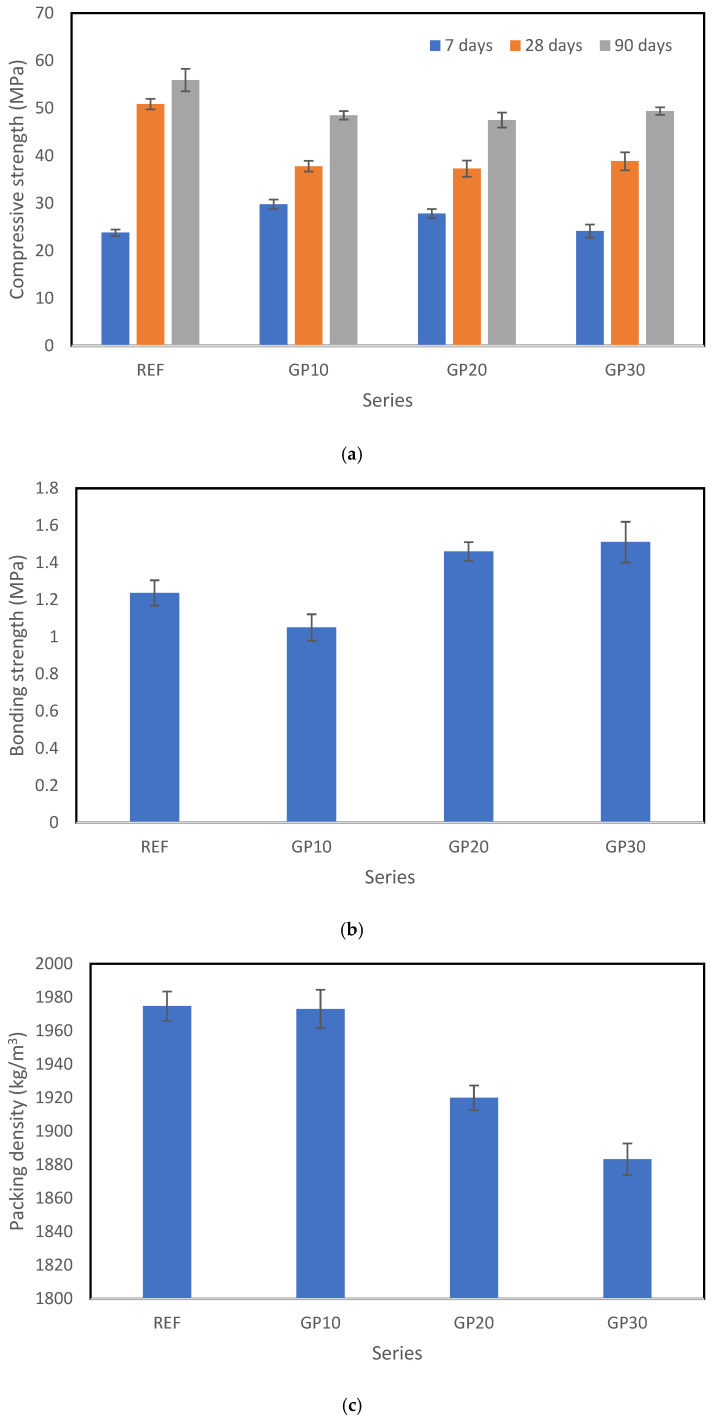
Results of experimental research obtained for each series: (**a**) compressive strength, (**b**) bonding strength and (**c**) packing density.

During the experimental program, five variables were evaluated. As mentioned above, these included compressive strength at three time intervals (7, 28, and 90 days), bonding strength measured using the pull-off method, and packing density. The results of these tests are presented in Figure 4, while Table 3 provides a statistical summary, including the mean, standard deviation, minimum, maximum, and coefficient of variation for the dataset.

**Figure 5:**
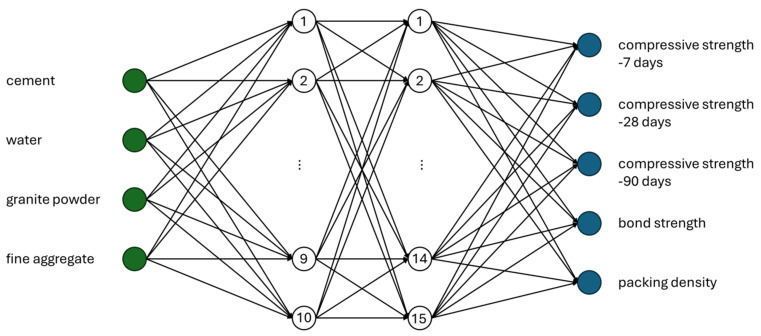
Scheme of the neural network used in the study.

**Figure 6:**
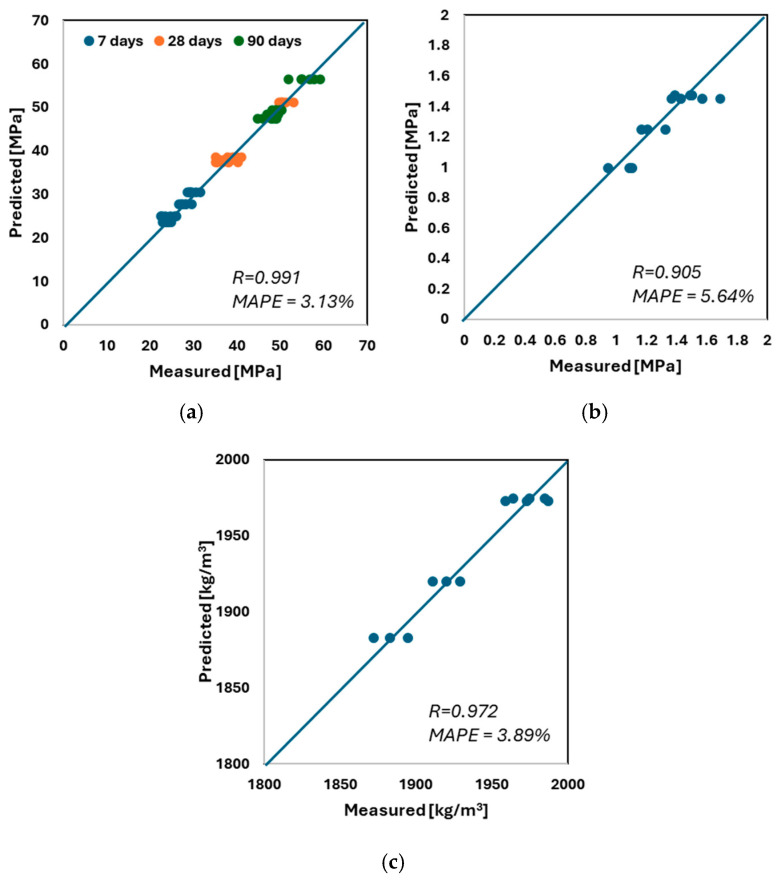
The relations between the measured and predicted values of (**a**) compressive strength, (**b**) bonding strength, and (**c**) packing density.

Analyzing the results presented in Figure 4 and Table 3, it can be observed that the data are mostly concentrated around the mean, as indicated by the relatively low standard deviation and coefficient of variation. From the compressive strength tests, it is evident that the increase in strength between 90 and 28 days is smaller than the increase observed between 7 and 28 days. When examining the strength parameters, it is also clear that in cases where the compressive strength after 28 days exceeds 35 MPa and the bonding strength is mostly above 1 MPa (with only one result below this threshold), these mixtures can be considered suitable for use in construction, as they meet the requirements of relevant standards. In the early stages, the compressive strength filling effect of the granite powder shows greater strength in comparison with the reference sample. Furthermore, in terms of late-stage compressive strength, it is noteworthy that the coefficient of variation is lower for the 90-day results compared to the 28-day results. This can be explained by the fact that while samples containing a high proportion of granite powder show significantly lower strength at 28 days, the difference becomes much less pronounced after 90 days. This experimental research aligns with the findings presented in [29]. As mentioned earlier, this study employed a Multilayer Perceptron Neural Network (MLP) for evaluation. The model was selected through an iterative process of increasing the number of hidden neurons in each layer, with the most accurate results obtained for a network consisting of four input parameters, ten neurons in the first hidden layer, fifteen neurons in the second hidden layer, and five output parameters. The schematic of this network is shown in Figure 5, while Figure 6 presents the prediction results for each property.

It can be seen from these charts that the model accurately predicts the values, as indicated by the very high linear regression coefficients (*R* > 0.9) and very low mean absolute percentage errors (*MAPE* < 6%) for all estimated variables. It is also evident that the model effectively categorizes the values, with no outliers observed.

## 4. Discussion

In this article, the authors present a numerical model employing a machine learning algorithm to predict five variables describing the physical and mechanical properties of cementitious composites containing granite powder. When compared with similar works in the field, the results obtained from the proposed model are unique in that they enable the prediction of more than one output property simultaneously. Furthermore, the accuracy of this model is demonstrated by the highly satisfactory values of the performance metrics. A comparison with similar studies from the literature, conducted by other researchers, is also provided in Table 4.

Analyzing the summary in Table 4, it is evident that the model developed in this study achieves a level of accuracy that is comparable to those proposed by other researchers for similar evaluations. However, although various assessment models have been presented, most predict only a single property, limiting their practical applicability. Furthermore, in most studies, the authors evaluated splitting tensile strength, which differs methodologically from pull-off strength. In contrast, when designing cementitious composite mixtures for flooring applications, assessing bonding strength using the pull-off method is more relevant and desirable than measuring splitting tensile strength.

## 5. Conclusions

In this article, a reliable model for predicting compressive strength, bonding strength, and packing density is presented. To achieve this, a dataset was developed based on an experimental program involving standardized testing of samples using established procedures. The material studied is a cement-based composite containing up to 30% granite powder. Based on the presented research, the following conclusions can be drawn:

- The model achieved very high values of the linear regression coefficient (*R* > 0.9) for all predicted properties, along with very low mean absolute percentage errors (*MAPE* < 6%).
- The most accurate model was developed using a multilayer perceptron (MLP) neural network, with input parameters including granite powder content, cement content, sand content, and water content. The network architecture consists of two hidden layers with 10 neurons in the first layer and 15 neurons in the second. The output parameters include early compressive strength (7 days), 28-day compressive strength, late compressive strength (90 days), bonding strength, and packing density.
- Utilizing granite powder, a waste product from rock cutting, offers a promising solution toward reaching Net Zero goals in the concrete industry. Since the granite powder used in this study is generated as a byproduct of the rock-cutting process, its associated CO_2_ emissions are attributed to that process. Therefore, incorporating it into concrete significantly reduces the overall carbon footprint of cement production.

The limitation of this model lies in the exclusive consideration of granite powder as a byproduct, with a maximum substitution level of 30% cement replacement. Future research could expand the dataset to include different waste-derived byproducts and higher substitution ratios, making the models more universal. Without such improvements and further experimental validation, it is not recommended to apply this model to other materials or mixtures with substitution levels outside of the studied range. Moreover, the successful implementation of a machine learning model predicting multiple output variables will likely increase the adoption of such models in data-driven concrete science studies.

## Figures and Tables

**Table 1:** Chemical composition of the materials used.

Material	CaO	SiO_2_	Al_2_O_3_	K_2_O	SO_3_	MgO	FeO	NaO	Fe_2_O_3_
Cement	56.62	16.03	6.1	7.32	6.62	2.61	2.26		2.44
Granite	8.3	53.63	19.9	3.29		5.54	3.46	3.46	2.42

**Table 2:** Material compositions of series.

Series [-]	Cement [kg/m^3^]	Water [kg/m^3^]	Granite Powder [kg/m^3^]	Fine Aggregate [kg/m^3^]
Ref	512.0	266	0.0	1536.0
GP10	460.8	266	51.2	1536.0
GP20	409.6	266	102.4	1536.0
GP30	358.4	266	153.6	1536.0

**Table 3:** Statistical description of the dataset used in the study.

	Compressive Strength 7 Days [MPa]	Compressive Strength 28 Days [MPa]	Compressive Strength 90 Days [MPa]	Bonding Strength [MPa]	Packing Density [kg/m^3^]
Mean	26.38	41.19	50.33	1.31	1937.76
Min	22.58	35.14	44.86	0.95	1872.00
Max	31.55	53.10	59.10	1.69	1987.00
Std.	2.72	5.80	3.64	0.20	39.48
CV	10.30	14.09	7.24	15.20	2.04

**Table 4:** Synthetic comparison of the models investigating the properties of granite-based materials.

Author	Predicted Properties	Model	Granite Usage	R, MAPE
Armaghani et al. [30]	Unconfined uniaxial compressive strength	ANN-LM 3-10-1	stone	R = 0.992, MAPE = 5.08%
Czarnecki et al. [31]	Compressive strength	RF	Cement substitutes max 30%	R = 0.989, MAPE = 3.30%
Fathy et al. [32]	Compressive strength	XGB	Cement substitutes max 9%	R = 0.999, MAPE = 0.5%
Chajec et al. [25]	Packing density	MLP 4-6-1	Cement substitutes max 30%	R = 0.956, MAPE = 0.582%
Endzhievskaya et al. [33]	Density	DT and RF	Microsilica substitute, Natural aggregate	MAE = 5.13—compression MAE = 33.37—density MAE = 0.36—bending
Moj et al. [34]	Bonding strength	RF	Cement substitutes max 30%	R^2^ = 0.944, MAPE = 3.23%
Rojo-López et al. [35]	Flow, Tfunnel	Genetic programming	Cement substitute up to 25%	R^2^ = 0.944—flow, R^2^ = 0.978—tfunnel
Czarnecki et al. [18]	Abrasion	NN and RF	Cement substitutes max 30%	R^2^ = 0.939, MAPE = 10.61%
Dafico et al. [36]	Moisture	NN	stone	R^2^ = 0.999, MAPE = 2.70%
This work	Compressive strength, bonding strength, packing density,	MLP 4-10-15-5		R = 0.991, MAPE = 3.13%,—compression R = 0.905, MAPE = 5.64%—bonding, R = 0.972, MAPE = 3.89%—packing density

## Data Availability

The original contributions presented in this study are included in the article. Further inquiries can be directed to the corresponding author.
